# Oligodendrocyte Precursor Cells Synthesize Neuromodulatory Factors

**DOI:** 10.1371/journal.pone.0127222

**Published:** 2015-05-12

**Authors:** Dominik Sakry, Hatice Yigit, Leda Dimou, Jacqueline Trotter

**Affiliations:** 1 Molecular Cell Biology, Department of Biology, Johannes Gutenberg University Mainz, D-55122 Mainz, Germany; 2 Physiological Genomics, Biomedical Center, Ludwig-Maximilians University Munich, D-80336 Munich, Germany; Hannover Medical School, GERMANY

## Abstract

NG2 protein-expressing oligodendrocyte progenitor cells (OPC) are a persisting and major glial cell population in the adult mammalian brain. Direct synaptic innervation of OPC by neurons throughout the brain together with their ability to sense neuronal network activity raises the question of additional physiological roles of OPC, supplementary to generating myelinating oligodendrocytes. In this study we investigated whether OPC express neuromodulatory factors, typically synthesized by other CNS cell types. Our results show that OPC express two well-characterized neuromodulatory proteins: Prostaglandin D2 synthase (PTGDS) and neuronal Pentraxin 2 (Nptx2/Narp). Expression levels of the enzyme PTGDS are influenced in cultured OPC by the NG2 intracellular region which can be released by cleavage and localizes to glial nuclei upon transfection. Furthermore PTGDS mRNA levels are reduced in OPC from NG2-KO mouse brain compared to WT cells after isolation by cell sorting and direct analysis. These results show that OPC can contribute to the expression of these proteins within the CNS and suggest PTGDS expression as a downstream target of NG2 signaling.

## Introduction

Oligodendrocyte progenitor cells (OPC) make up at least 5% of total cells in all regions of the developing and adult mouse CNS [1]. They are migratory, proliferative [2–5] and can differentiate into myelinating oligodendrocytes [6–8] in both development and disease [9–11]. A large fraction of OPC however remains as a self-renewing population throughout the adult brain [3]. Synaptic innervation from neurons has been shown by excitatory (glutamatergic) and inhibitory (GABAergic) synapses in the hippocampus [12–15] and furthermore been demonstrated in cerebellum, corpus callosum and the cortex [16–18]. Initial studies postulated that these synapses allow OPC to respond to neuronal activity regulating cell differentiation, synthesis of Myelin Basic Protein and thus the initial steps of the myelination process [3, 19]. Additionally positioning and migration of OPC during development appears to be synaptically controlled [20]. However, a potential role of this innervation allowing OPC in the CNS to signal back to neurons, independent of their differentiation to myelinating cells, has recently been described, where the LNS domains of the ectodomain released from the OPC protein NG2 modulate neuronal glutamatergic signaling [21]. Expression of additional neuromodulatory factors by OPC would increase the spectrum of mechanisms used by OPC to signal to the neuronal network [22].

Expression of the chondroitin sulfate proteoglycan type-1 membrane protein NG2 (CSPG4) is characteristically used to identify OPC which are further defined by PDGFR-α expression in development and in the adult [23–27], as it is not expressed by neurons or other glia. NG2 is a large protein of around 300 kD (full-length, FL) with a small intracellular region of 8.5 kD. The creation of a ~12 kD NG2 membrane bound C-terminal fragment (CTF) after release of the 290 kD ectodomain generated by α-secretase cleavage from the NG2 FL protein was implied [28, 29] and recently directly demonstrated [21]. The intracellular region can be released in a γ-secretase-dependent mechanism from the NG2 (CTF), after initial α-secretase processing of the full-length protein [21].

The intracellular region of NG2 cleaved from the CTF is referred to as NG2 ICD, in analogy to the ICDs of other proteins such as Notch [30, 31]. Intracellular NG2 interaction partners have been identified for the C-terminal PDZ binding motif: these are GRIP, Mupp1 and Syntenin [32–34]. Two tyrosines (Tyr9292/030) are targets for PKCα and the ERK kinases [35], the latter pathway has been shown to influence OPC migration in a growth factor-dependent manner [2].

Here we show that OPC express two known neuromodulatory proteins, Prostaglandin D2 synthase (PTGDS) and neuronal Pentraxin 2 (Nptx2/Narp). Primary cultured OPC express the proteins in a differentiation-dependent manner. The expression of PTGDS is influenced by the NG2 CTF and ICD, the latter is predominantly localized in the nucleus of OPC upon expression of transfected constructs. Interestingly, in FACS-sorted cells from P9 mouse brain, PTGDS but not Nptx2 mRNA is highly reduced in OPC derived from NG2-KO mice compared to OPC isolated from WT animals. This is compatible with reduced PTGDS protein levels in cultured OPC after NG2 knock-down by siRNA. Our results show that *in vivo* OPC contribute to the expression levels of the neuromodulatory factors PTGDS and Nptx2 and further suggest that PTGDS is a target of NG2 signaling in OPC.

## Materials and Methods

### Cell lines

The OPC cell line Oli-neu (as established in [36]) was cultured on PLL coated dishes in SATO medium with 1% Horse Serum (HS). HEK 293 (HEK, Invitrogen) cells were cultivated in DMEM (Sigma) with 1% pyruvate 10% FCS.

### The NG2-EYFP knock out mouse

Homozygous NG2-EYFP mice (NG2^-/-^), lack NG2 protein expression, referred to as NG2-KO (knockout), and were previously described [37]. All animal experiments were carried out in strict accordance with protocols approved by local Animal Care and Use Committee of Johannes Gutenberg University of Mainz. Mice were sacrificed by decapitation to remove the brain.

### Isolation of primary OPC by magnetic or fluorescence-activated cell sorting

Single cell suspensions were obtained from total brains of Postnatal day (P) P9 C57Bl/6N mice and NG2-KO mice using the NTDK-P Kit (Miltenyi Biotec). Magnetic isolation (MACS) was performed as described previously [38] from wild-type (WT) animals with anti-NG2 beads from the same vendor according to the Miltenyi protocol. Cells were cultured at 150.000 cells/well in 24-well plates in SATO + B27 supplement with 2mM L-glutamin, PDGF-AA (10 μg/ml) and FGF-2 (5 μg/ml). Cells or cell lysates were prepared and analyzed at the following times after plating the cells: 2hours (h) / 0 days in vitro (DIV0), 24h / DIV1, 48h/ DIV2, or 96h/ DIV4. Isolation of cells by fluorescent activated cell sorting (FACS) was performed in the FACS core facility (IMB Mainz). For each sort, 3–4 P9 brains were pooled. PDGFR-α antibody staining and endogenous EYFP labeling were used to sort OPCs from WT and NG2-KO animals respectively. 500,000 cells (from the positive and negative sort fractions) were used for total RNA isolation.

### Transient transfection of cells

The OPC cell-line Oli-neu was transfected using polyethylenimine (PEI, 1 μg/μl, Sigma) at a ratio of 1:4 (DNA:PEI; w/v). Transfection solution was applied to cells after 16h of culture. Adherent primary OPC (pOPC, isolated by MACS) were transfected in single wells of a 24-well plate with the AMAXA 4D Y-Unit, program ED158, 2h after plating with 30 μg DNA per well. pOPC were fixed 24h after transfection.

The following plasmids were used: NG2_del (pRK5, mouse cDNA, AA 1–477_Δ_2206–2327); NG2_ICD (pRK5, mouse cDNA, AA 2251–2327); NG2_del_Myc (pcDNA4); NG2_ICD_Myc (pcDNA4); H2B-GFP [39].

siRNA was obtained from Qiagen and Oli-neu cells in suspension were transfected with the AMAXA nucleofector II system using the primary neural cell protocol. The targeted sequences of mouse cDNA were as followed: siNG2 (5’-AAGTCAGCTCACTGCAGAAAA-3’); The non-silencing siRNA (siC) (5’-AATTCTCCGAACGTGTCACGT-3’) was used as a control.

### Primary antibodies

The following primary antibodies (AB) were used for Western Blot and immunostainings: NG2 monoclonal (mc), rat, [40]; NG2 cytoplasmic (cyto), rabbit, [41]; NG2 polyclonal (pc), rabbit, [40]; GAPDH, rabbit, (Bethyl); Myc, mouse, (Sigma); α-Tubulin, mouse, (Sigma); ADAM 10, rabbit, (Abcam); PTGDS, rabbit, (Abcam and Abnova as used in [42]); Nptx2, rabbit, (Abcam) Olig2, rabbit, (Millipore); PDGFRα, rat, (Becton Dickinson); GFAP, rabbit, (Dako); F4/80, rat, [43]; TUJ-1, rabbit, (Covance); PLP (clone aa3), rat; SMA, mouse, (Sigma).

### Soluble protein fraction from total fore-brain

P9 mouse brains of NG2-KO and WT (the same age used for FACS and MACS sorting) were prepared in ice-cold HBSS. The cerebellum and olfactory bulb were removed from the separated hemispheres and the forebrains were shock frozen in liquid N_2_. Tissue was homogenized in Buffer A (0.32 M sucrose, 5 mM HEPES, protease inhibitors, pH 7.4) at 10 ml / g, with a Potter S (12 strokes, 900 rpm), followed by differential centrifugation. The nuclei-free supernatant (generated by centrifugation at 1,000 g, 10 min, 4°C) was further centrifuged for 20 min at 12,000 g and 4°C. The resulting supernatant was defined as the soluble protein fraction.

### Postnuclear cell lysates

Cells were washed with PBS and scraped with a rubber policeman into lysis buffer (PBS, 1% TX-100, protease inhibitor cocktail (Roche complete)) from the culture plate. After incubation for 30 min at 4°C and centrifugation at 1000g, 10 min, 4°C, supernatants were defined as postnuclear lysates (PN). The same volume of lysis buffer was used per sample and equal volumes loaded for WB.

### SDS-PAGE and Western Blotting

Equal volumes of protein solution or amounts of total protein were used for analysis. Protein concentration was determined by the BCA protein assay (Pierce) or OD_280_. All samples were diluted with 1-4x SDS or LDS (Invitrogen) sample buffer, heated to 80°C for 10 min and resolved on 4–12% NuPage BisTris gradient gel in combination with MES or MOPS running buffer (Invitrogen). Western blotting was done with the respective NuPage Blot system utilizing a PVDF membrane (Millipore). The latter was blocked in PBS containing 4% nonfat milk and 0.1% Tween 20. Signal detection was carried out using HRP-conjugated secondary antibodies (Dianova), enhanced chemiluminescence (ECL) assay solution (Millipore) and hyperfilms (GE). ImageJ 1.46 (NIH) was used for signal quantification and all protein levels were normalized against those of GAPDH from the same sample.

### Quantitative real time PCR

Total RNA was purified from cultured cells with the RNeasy Mini Kit (Qiagen). Concentration was determined with a NanoDrop photometer (PeqLab). cDNA synthesis was performed with the first strand cDNA synthesis Kit (Roche) using random hexamer primers. Quantitative real time PCR was performed in a StepOne cycler using the TaqMan system (AppliedBiosystems). All targets were normalized against GAPDH (ΔCT = (CT target)–(CT GAPDH)). For ΔΔCT see individual figure legends. All ΔCT and ΔΔCT values are log2 scale.

### Immunocytochemistry

Cells were cultured on poly-L-Lysine (PLL) coated coverslips, washed with PBS and fixed with 4% PFA for 15 min at RT, washed and permeabilized for 2–5 min with 0.1% TX-100. Blocking was with 10%HS for 15 min. Primary and secondary AB were applied for 30 min at RT. Coverslips were mounted in Moviol. Primary AB were used as described above. Secondary AB: goat or donkey anti mouse, rat, rabbit or guinea pig coupled with Alexa488, 546, 647 or Cy3, Cy5 (Invitrogen, Dianova). DAPI (0.1μg/ml) was added together with the secondary AB. Images were taken with a Leica DM6000 fluorescent microscope or a SP5 laser scanning confocal microscope (IMB Mainz). Image processing was done with Leica LAS AF and ImageJ (NIH), specifically the ImageJ3DViewer [44].

### In situ hybridization

In situ hybridization was carried out according to the manufacturer’s procedure (ViewRNATM ISH Cell Assay, Affymetrix), without Proteinase K treatment as reported in [45]. Cells were fixed by 4%PFA at RT for 10 min, permeabilized and hybridization was performed using probes recognizing PTGDS and Nptx2. After incubation with labelled probes, mRNA localization was combined with immunocytochemical staining for NG2 (1:50 for MACS sorted cells, 1:100 for Oli-neu cells).

### Statistics

Statistical analyses were performed using the two tailed student’s t-test or the Wilcoxon—Mann—Whitney test, with MS Excel or SPSS. Significance was classified as follows: *, n ≤ 0.05; **, n < 0.01; ***, n < 0.001. For all mean values SEM is shown.

## Results

### Expression of PTGDS and Nptx2 by cultured primary OPC

For a related project, a stab wound in the cerebral cortical gray matter was performed from NG2-knockout (KO) and WT mice. Lesioned tissue (included tissue in a radius of 350μm around the lesion but excluding the white matter) containing all cell-types, was taken from each genotype 3 days after injury. The proteins PTGDS [46] and Nptx2 [47] were selected from an (unpublished) mRNA microarray screen from these tissues, as the two sole neuromodulatory target-genes differing (≥ 2-fold change) in expression level from total tissue samples of lesioned wildtype (WT) compared to lesioned NG2-KO adult mice. PTGDS is the key enzyme in prostaglandin D2 signaling, converting prostaglandin H2 to D2 and has been reported to be mainly expressed by mature oligodendrocytes [46, 48]. Nptx2 has been reported to be secreted by neurons in an activity-dependent fashion, binding to extracellular neuronal AMPA receptors and thereby influencing their surface stability and trafficking [49]. To investigate if OPC express these proteins in the intact nervous system and if NG2 (-cleavage) could contribute to possible differences in the expression profiles under non-lesion conditions, the following experiments were performed.

**Fig 1:**
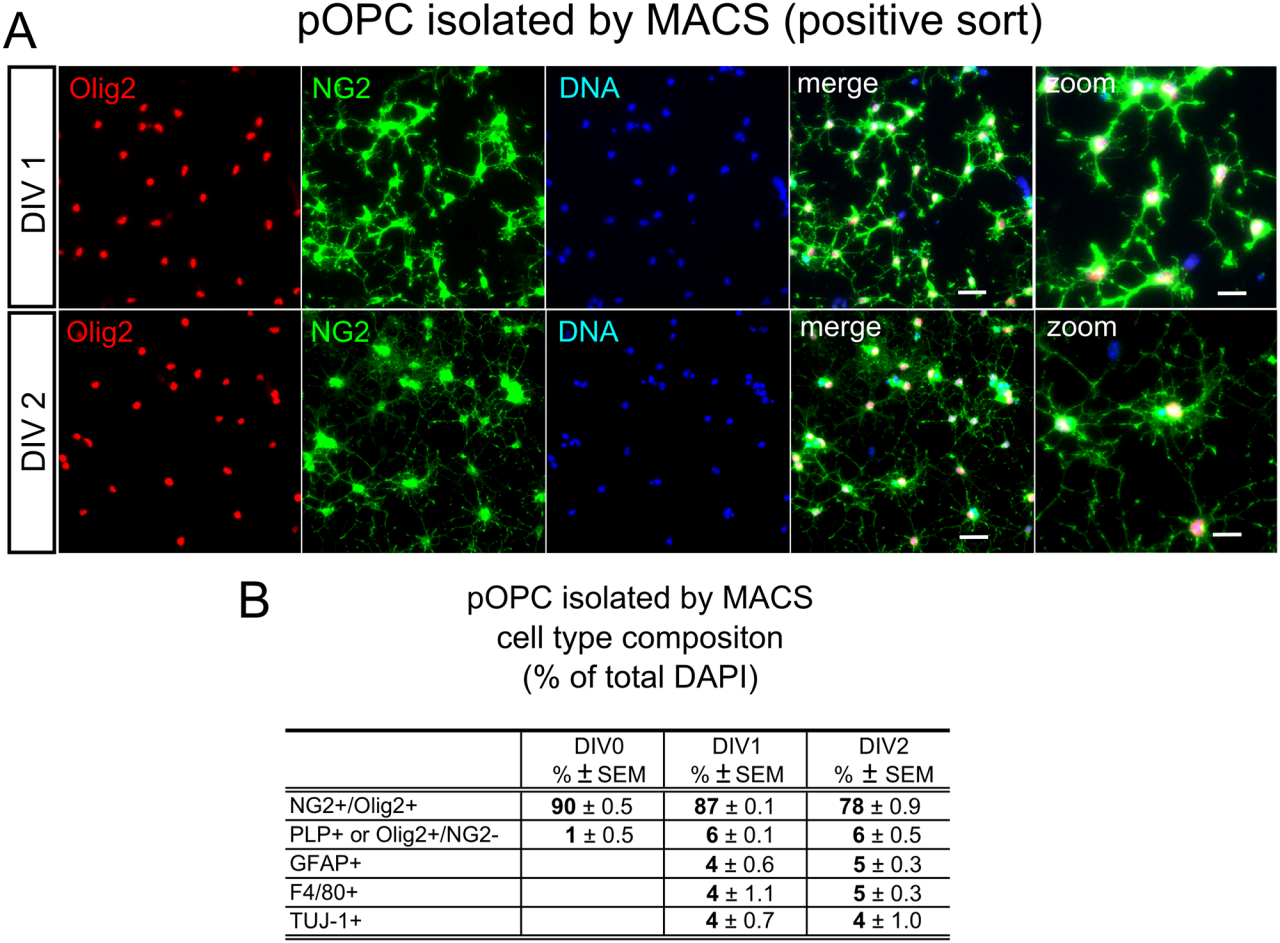
Primary OPC culture. **A** Cell-type specific staining of magnetically sorted (MACS) primary OPC (pOPC) at different timepoints of culture. DIV0, 1 and 2: 2h, 24h and 48h of culture respectively (DIV: days in vitro). OPC were identified by co-expression of NG2 and Olig2 (NG2+/Olig2+), Scale bar = 30μm and 15μm for the zoom. The percentage of the identity of each cell-type of the total DAPI+ cells is given in **B**. Differentiated oligodendrocytes were identified by the expression of PLP, astrocytes by expression of GFAP, microglia by expression of F4/80 and neurons by expression of ß-III-tubulin (TUJ-1). Pericytes were not detected in in the pOPC culture, see S1 Fig for pericyte staining. (A&B: 200 cells were analyzed for each staining/time-point from 2 independent MACS sorts).

**Fig 2:**
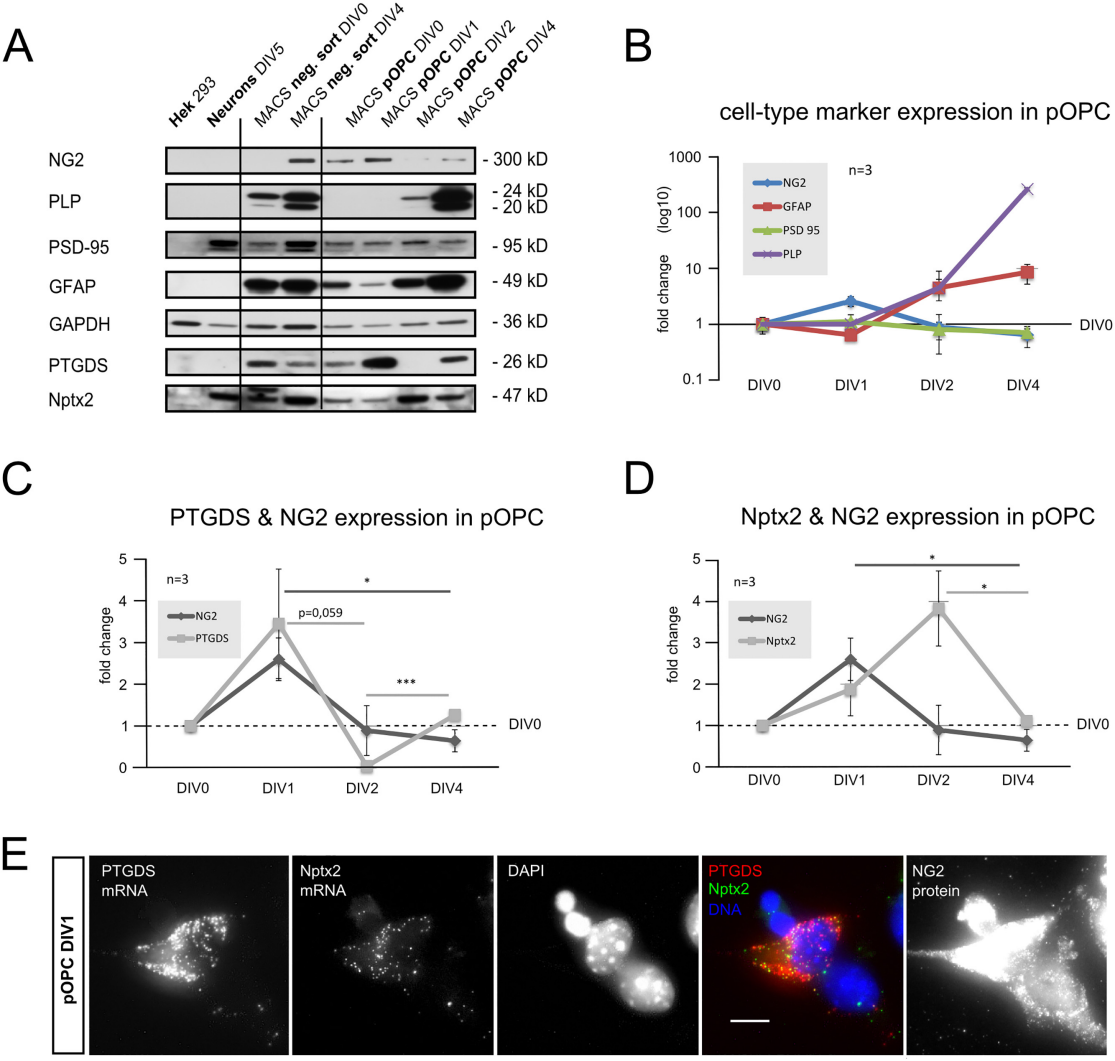
Expression of PTGDS and Nptx2 in primary OPC. **A** Expression of cell-type specific markers was analyzed by Western-Blot of total cell lysates of pOPC and the negative (neg.) sort fractions, HEK cells and primary cortical neurons (DIV5 in culture). **B-D** show the expression of proteins in pOPC over time, related to DIV0. In **B** a peak of the OPC protein NG2 is shown at DIV1, PLP indicates differentiation into oligodendrocytes starting at DIV2, GFAP shows astrocyte differentiation starting at DIV2. **C** PTGDS expression peaks together with NG2 at DIV1. **D** Nptx2 expression increases together with NG2 at DIV1, peaks at DIV2 and returns to basal levels at DIV4. **E** Expression of PTGDS and Nptx2 mRNA in pOPC at DIV1, as revealed by *in situ* hybridization, OPC were identified by antibody staining of NG2 protein. (A-D: 3 sorts were analyzed for each time point. Two tailed student’s t-test was applied.)

We first analyzed the differentiation-dependent expression of these two proteins in cultured primary OPC (pOPC) isolated from postnatal day 9 mouse brains (P9) by magnetic cell sorting (MACS). It has been described previously that pOPC isolated with this technique differentiate with time (4–6 days in vitro; DIV4-6) into oligodendrocytes and astrocytes [38], however OPC make up the large majority (80–90% of all DAPI+ cells) during the first two days of culture (Fig 1A and 1B). We also observed a small contamination with non-OPC lineage cells such as astrocytes (GFAP+), microglia (F4/80+) and neurons (TUJ-1+) of around 4% for each of these cell-types. We investigated possible contamination of the OPC fraction with neurovascular cells expressing NG2 (pericytes), by searching for cells double-stained with NG2 and smooth muscle actin (SMA). These were present in primary mixed cultures (S1 Fig), but absent from the positive sorted fraction of OPC (pOPC). Control stainings of pOPC and mixed primary cultures for all neural and pericyte markers used are shown in S1 Fig. Expression of cell-type markers and the proteins of interest is shown in the Western Blot in Fig 1C. pOPC have been analyzed at DIV0, 1, 2 and 4 and the negative (neg.) sort at DIV0 and 4. As positive and negative controls, lysates of primary cortical neurons at DIV5 and from the non-neuronal cell line HEK, were used. In Fig 1D the expression of cell-type markers in pOPC is presented, dependent on their time in culture, showing a peak of the OPC protein NG2 at DIV1. PLP, as a marker for mature oligodendrocytes, is not detectable until DIV2, demonstrating that at the first two time-points the population consists of undifferentiated OPC as expected. GFAP, as a marker for astrocytes, is at a low level at the first two time points and increases at later stages. PSD-95 was used as a typical neuronal postsynaptic marker in all experiments, and shows a low stable level in the pOPC culture, in contrast to the other cell types (Fig 2A).

As reported above, OPC are the major cell-population (90–80%) until DIV2 in culture. NG2 protein levels, as a marker for OPC, peak at DIV1 with similar levels at DIV0 and 2 (Fig 2B–2D). We observed that the two analyzed neuromodulatory proteins exhibited peak protein levels within this time window where the cultures contain predominantly OPC. PTGDS protein levels peak, like those of NG2, at DIV1. At DIV2 PTGDS protein is at very low levels and returns to the starting level at DIV4 (Fig 2C), suggesting that PTGDS is highly expressed in OPC expressing high levels of NG2 protein and re-expressed by maturing oligodendrocytes. Neurons and HEK cells showed no expression of PTGDS protein at any time point studied (Fig 2A). Nptx2 protein levels peak at DIV2 and then decrease to starting levels at DIV4 (Fig 2D), showing an expression maximum at a time point where the cultures contain predominantly OPC and suggesting a decreased expression with ongoing maturation of OPC. Levels of Nptx2 protein were similar in DIV5 primary neurons and pOPC at DIV2, while the protein was absent from HEK cells (Fig 2A). mRNA for PTGDS and Nptx2 was detected by *in situ* hybridization in subpopulations of pOPC, identified by expression of the NG2 protein by antibody staining (Fig 2E).

### NG2-dependent in vivo expression of PTGDS and Nptx2

**Fig 3:**
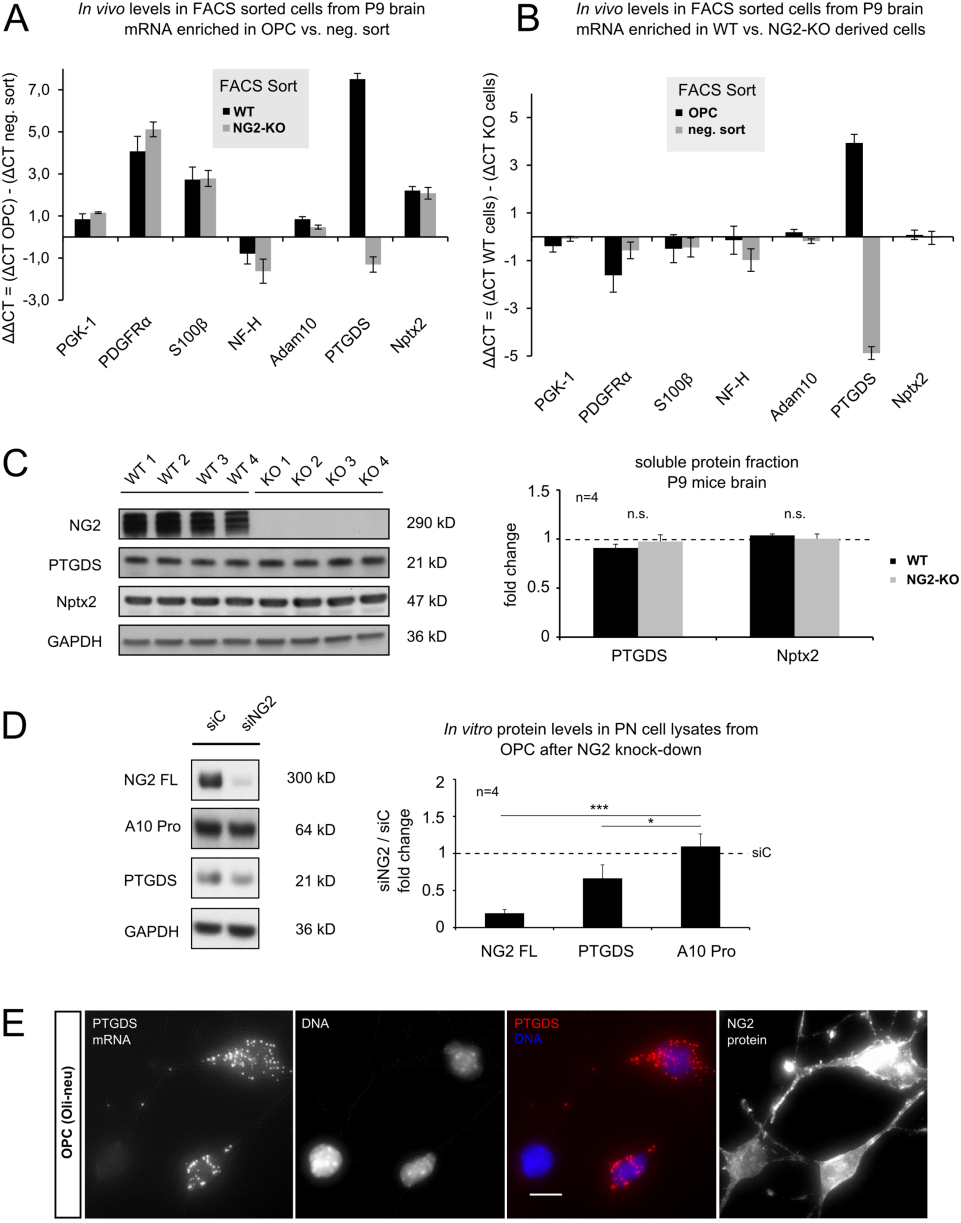
NG2 dependent regulation of PTGDS and Nptx2 *in vivo*. **A&B** mRNA levels of target-genes were directly analyzed by qRT-PCR from FACS isolated OPC and other cells (negative (neg.) sort). Single cell suspensions from total brains of postnatal day 9 (P9) NG2-KO and WT mice were used for FACS. **A** Enrichment of target mRNA in OPC in comparison to other cell-types is shown for WT (black bar) and NG2-KO (grey bar), (ΔΔCT = [ΔCT OPC]–[ΔCT other cells]). Enrichment of PDGFRα mRNA validates OPC enrichment. Nptx2 mRNA was enriched within OPC of both genotypes, while PTGDS mRNA was only enriched in WT OPC. **B** Genotype-specific mRNA enrichment in OPC (black bar) and other cells (grey bar) is plotted (ΔΔCT = [ΔCT WT cells]–[ΔCT KO cells]). PTGDS was the sole target gene analyzed exhibiting differential expression between WT and KO genotypes. Expression was highly increased in WT-derived OPC and down-regulated in the other cells (neg. sort) from these mice. **C** Western Blot of soluble protein fractions. Soluble fractions were extracted from P9 mouse brain of WT and NG2-KO mice. Total PTGDS and Nptx2 protein levels show no difference between genotypes. **D** Protein levels of post nuclear (PN) cell lysates of the OPC cell line Oli-neu were analyzed after treatment with siRNA silencing NG2 expression (siNG2) or control siRNA (siC). Full-length NG2 levels were reduced as well as PTGDS protein levels, fitting to the reduced mRNA levels of PTGDS found in NG2-KO OPC (B). **E** Expression of PTGDS mRNA by the OPC cell-line Oli-neu as revealed by *in situ* hybridization. (A&B: for NG2-KO OPC, 4 independent sorts were analyzed, for WT 3 sorts were analyzed, ΔCT = (CT target)–(CT GAPDH), ΔCT and ΔΔCT values are in log2 scale; for **C:** 4 animals were analyzed for NG2-KO (KO1-4) and BL6/N (WT1-4) mice; for **D**: 4 independent transfections were analyzed per siRNA, two tailed student´s t-test was performed.)

To investigate potential NG2 protein-derived effects on expression of PTGDS and Nptx2 *in vivo*, we analyzed mRNA levels of OPC (positive sort) directly after FACS sorting from postnatal (P9) NG2-KO and WT mice brain by qRT-PCR. The negative sort fraction containing other cell types (neg. sort) from both genotypes was also analyzed. Fig 3A shows enrichment of selected mRNAs in OPC versus neg. sort for WT (black bar) and NG2-KO (grey bar). PDGFR-α, a classic OPC marker, is-as expected- highly enriched in the OPC population (ΔΔCT of 4), with no difference in enrichment level between the genotypes. In contrast, Nptx2 mRNA was enriched 4-fold (ΔΔCT of 2) in OPC versus cells in the neg. sort, validating an expression by OPC. PTGDS was the only gene examined where the mRNA was enriched differently between the WT and NG2-KO genotypes: here a striking enrichment of PTGDS mRNA was found in WT OPC compared to the WT neg. sort (ΔΔCT of 7), while in OPC from NG2-KO mice a lower level was observed compared to the NG2-KO neg. sort fraction (ΔΔCT of -1).

To better illustrate genotype-specific expression, mRNA levels were compared in WT and NG2-KO cells (Fig 3B), both for the OPC (black bar) and the neg. sort (grey bar). Here Nptx2 showed no difference in expression between the genotypes in either fraction, similar to all other targets analyzed. However PTGDS mRNA expression levels were strongly increased in WT OPC compared to NG2-KO OPC (ΔΔCT of 4), while they were strongly decreased in the negative sort fraction from WT animals (ΔΔCT of -4). These results suggest a compensatory regulation of PTGDS expression by another cell type present in the cell fraction from the negative sort. This was further validated by analyzing protein levels of the soluble brain fraction from P9 mice. This showed no significant difference between the two genotypes (Fig 3C).

We further investigated whether the silencing of NG2 influences PTGDS protein levels in the OPC cell line (Oli-neu) (Fig 3D). NG2 protein expression was silenced by transfection with a specific siRNA (siNG2) and a non-specific siRNA as a control (siC). In line with the reduced PTGDS mRNA levels we found in FACS-sorted OPC from NG2-KO mice brains (Fig 3B), silencing of NG2 in Oli-neu cells significantly reduced the PTGDS protein levels (Fig 3D). Expression of the non-proteolytic active form (Pro form) of ADAM 10 (A10 Pro) was used as a control type-1 membrane protein: this was not affected by silencing of NG2 protein expression. In accordance with these findings a subpopulation of Oli-neu cells also expressed PTGDS mRNA by *in situ* hybridization (Fig 3E).

### The NG2 ICD localizes to the cell nucleus

**Fig 4:**
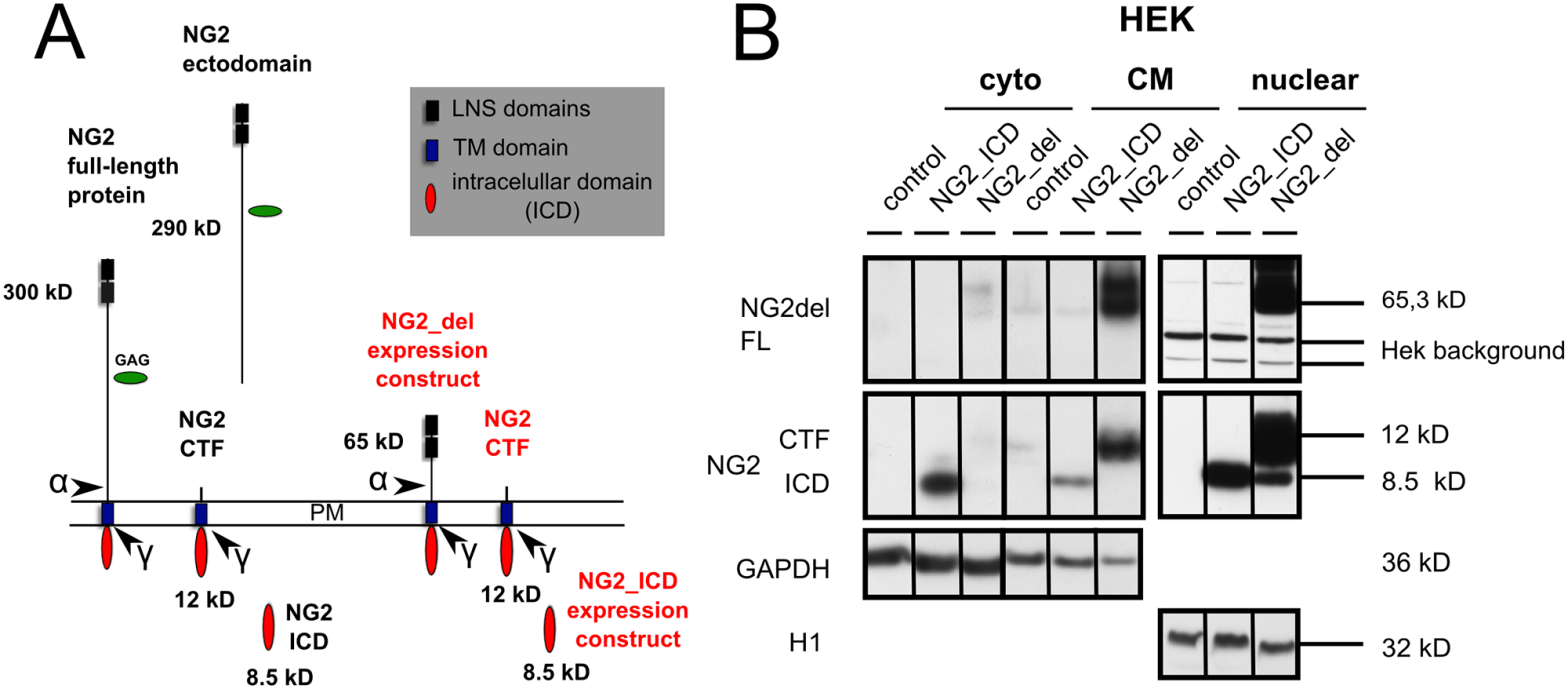
The NG2 ICD is located in the nucleus. **A** Cartoon of the NG2 full length protein and the major cleavage fragments (ectodomain, CTF, ICD). Ectodomain cleavage (indicated by the α) has been reported by others [28], while intracellular cleavage (indicated by the γ) has been found by our group [21]. Expression constructs NG2_del leading to high NG2 CTF and lower ICD levels by proteolytic processing and the NG2_ICD construct leading to high levels of NG2 ICD are both shown in red. **B** Cytoplasmic (cyto), crude membrane (CM) and nuclear fractions of HEK cells transfected with empty plasmid (control), NG2_ICD, or NG2_del are shown. NG2_del full-length (FL) protein, the membrane bound NG2 CTF and the NG2 ICD are shown in WB (schematically shown in Fig 2D). The NG2 ICD shows the highest levels when expressed as a recombinant protein (NG2_ICD). This is present in all fractions but highest in the nuclear fraction. NG2_del-derived NG2 ICD (generated by proteolysis) is only detectable in the nuclear fraction and runs at the same size as the recombinant NG2 ICD.

Nuclear translocation and signaling of the NG2 intracellular domain (ICD), in a similar fashion to the signaling properties of the Notch ICD (NICD), may provide a mechanism for NG2 to modulate PTGDS expression. We previously showed [21] that the NG2 ICD can be released by intracellular cleavage from the NG2 CTF (Fig 4A). The NG2 CTF results after NG2 ectodomain cleavage as reported by others [28], again analogous to the cleavage of the Notch protein [30, 50].

To study the role of the NG2 ICD we first utilized a recombinant NG2 ICD protein (NG2_ICD, Fig 4A) of similar size to the endogenous ICD and analyzed whether it localizes to the nucleus. Additionally, we used recombinant membrane-bound truncated NG2 (NG2_del, Fig 4A), which leads to high levels of NG2 CTF and low NG2 ICD levels ([21] and the experiments performed in HEK cells are shown below). In biochemical fractions of HEK cells (these have no endogenous NG2) transfected with plasmids coding for untagged NG2_ICD or NG2_del constructs, levels of NG2 ICD protein were highest in nuclear and cytoplasmic (cyto) fractions compared to the crude membrane (CM) fraction. Nuclear levels of NG2 ICD after NG2_ICD transfection were much higher than those of nuclear NG2 ICD in NG2_del transfected cells (Fig 4B). In the case of NG2_del, low levels of the ICD can be generated by proteolytic cleavage of the NG2 CTF and are of the same molecular weight as those of the recombinant NG2_ICD (Fig 4A and 4B nuclear fraction).

**Fig 5:**
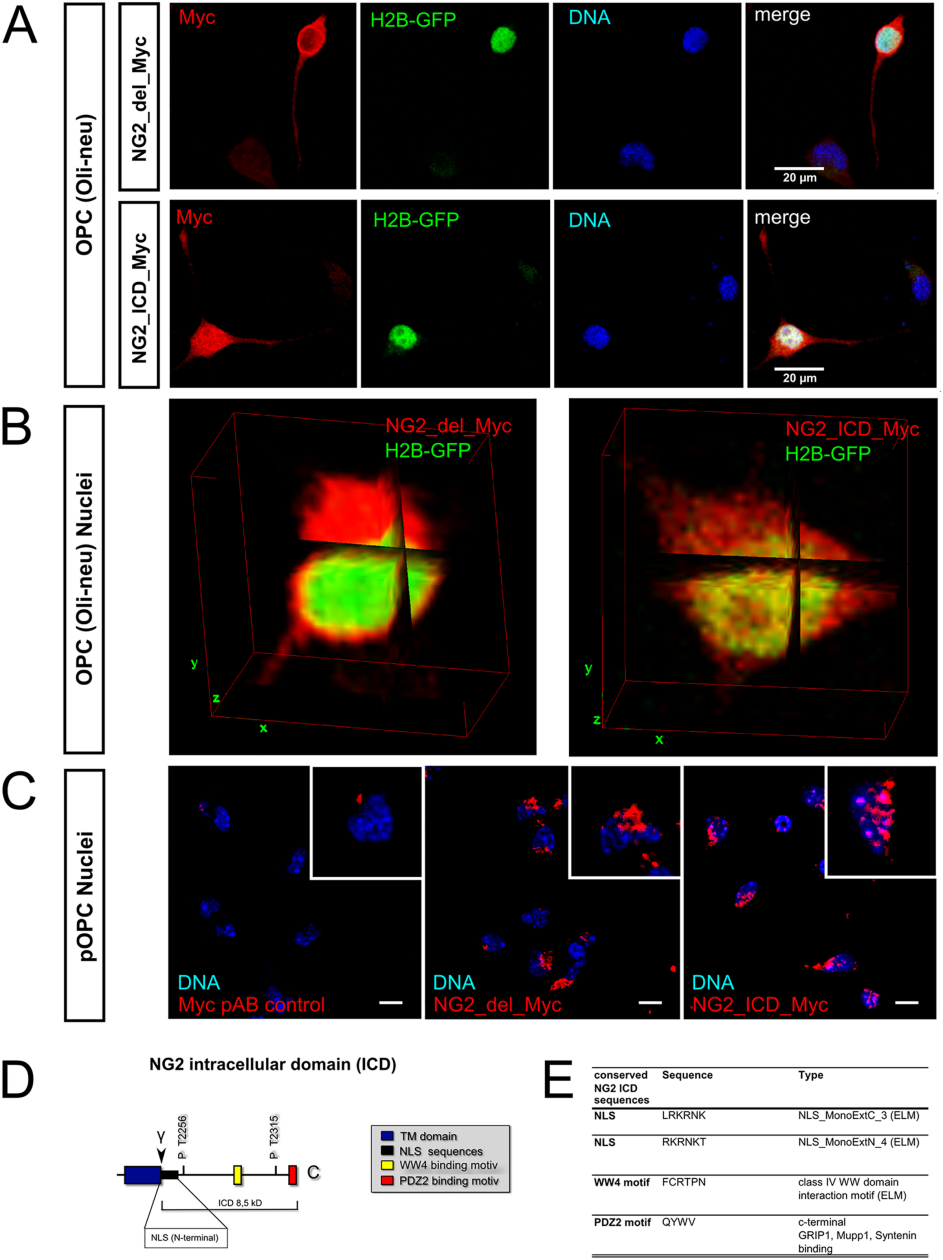
NG2 fragments exhibit distinct subcellular localization including the nucleus. Fluorescent staining of OPC expressing NG2_del_Myc or the NG2_ICD_Myc construct (in red) and GFP tagged Histone2B (H2B-GFP, only A&B). **A&B** Confocal laser scanning microscope (cLSM) pictures of Oli-neu (OPC cell line) show NG2_del_Myc (red) staining at the plasma membrane and intracellular membranes, but nuclei are largely spared. Expression of the NG2 ICD (NG2_ICD_Myc, red) results in an almost homogeneous cytoplasmic staining including an intense staining of the nuclei. **A** Standard deviation (STD) projection is shown for the total LSM stack. NG2 ICD (Myc staining) shows much stronger overlap with nuclear stainings (H2B-GFP, DAPI) than the one of NG2_del (scale bar = 20μm). **B** Three dimensional orthogonal views (ImageJ3DViewer) are shown for cell bodies of the two transfection conditions. NG2 ICD reveals a striking intranuclear staining (overlap with H2B-GFP), while NG2_del only overlaps at the outer layer of the H2B stained nucleus. **C** Transfected pOPC showed a much lower expression of both NG2 constructs. The strongest staining of Myc-tagged constructs is observed close to the nucleus in NG2_del_Myc transfected cells. In NG2_ICD_myc transfected cells, many cells additionally show a staining of nuclear substructures. Images show one plane from the center of a confocal z-stack (pAB = primary antibody, scale bar = 15μm). For staining of pOPC also see S2B Fig. **D** Cartoon of the intracellular domain (ICD) of NG2. **E** Selected NG2 ICD sequences with predicted functions. ELM database analyses show 2 NLS sequences indicating nuclear transport and a predicted WW4 binding motif, a binding site for protein complexes which localize to nuclei. (A&B: 20 double-transfected cells from 3 independent transfections per condition were analyzed; 100% of the NG2_del and over 70% of the NG2_ICD transfected cells showed the described effects. cLSM stacks had a z-stack depth of at least 12 μm, with a z-increment of < 0.3 μm per single image.)

In Oli-neu cells transfected with NG2_del with an additional C-terminal Myc tag (NG2_del_Myc), a strong staining of Myc at the plasma membrane and in intracellular membranes was observed, but the cell nuclei were almost completely unstained (Fig 5A and 5B). In contrast, transfection of a plasmid coding for the NG2 intracellular domain (NG2_ICD) with a C-terminal Myc tag (NG2_ICD_Myc), showed an almost homogeneous intracellular staining for Myc which included the nuclei, as shown by an overlap with DAPI staining (for DNA) and GFP-coupled histone 2B (H2B-GFP) (Fig 5A and 5B).

Similar results were observed in primary OPC isolated by MACS, although the transfection efficiency was lower and Myc signals were weaker compared to Oli-neu cells. After subtraction of the signal from the primary myc AB control from the GFP transfected control (pAB control), only a staining close to the nucleus remained in the case of NG2_del_Myc expressing cells. In contrast, in cells transfected with NG2_ICD_Myc plasmids a Myc staining within the nucleus was observed, especially in areas of high DNA concentration indicated by brighter DAPI staining (Fig 5C). An overview of transfected and stained pOPC is shown in S2 Fig.

To identify sequences on the NG2 ICD which could support a nuclear localization, we performed an ELM database (elm.eu.org) analysis of the ICD amino acid sequence. Interestingly we detected two predicted Nuclear Localization Sequences (NLS) as well as a WW4 binding motif, suggesting an interaction with WW4 domain-containing proteins (Fig 5D and 5E). The PDZ2 binding motif has already been described and several binding partners have been identified.

### NG2 fragments influence PTGDS protein expression

**Fig 6:**
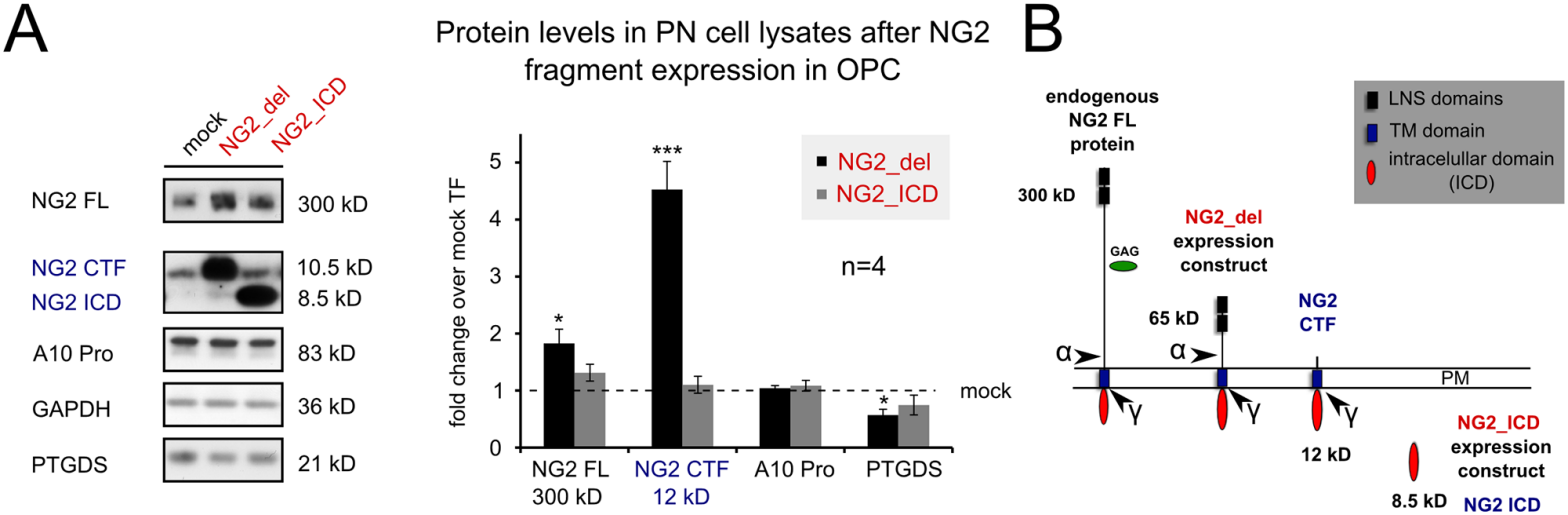
NG2 intracellular fragments influence PTGDS protein levels. **A** Expression of NG2_del, NG2_ICD (both red) and Mock (empty Plasmid) constructs in the OPC cell-line Oli-neu, resulted in a reduction of PTGDS protein levels in post nuclear lysates (PN). Overexpression of these constructs leads to a changed ratio of protein levels between the NG2 FL and the small NG2 (intracellular) cleavage fragments the CTF and the ICD (compare to mock, see B). **B** Cartoon of NG2, NG2 expression constructs (red) and the cleavage sites for α- and γ-secretase leading to the creation of the CTF (12 kD) and ICD (8.5 kD, both blue). (A: 4 independent transfections were analyzed per construct; two tailed student’s t-test was performed.)

The data shown above suggest that the NG2 ICD could have a nuclear signaling function. We therefore investigated whether expression of the truncated membrane bound NG2_del or the soluble NG2_ICD expression construct (Fig 6B, red text) could influence protein expression levels of PTGDS in the OPC cell line Oli-neu, as PTGDS expression is influenced by a reduction of total endogenous NG2 protein levels in the NG2-KO OPC, or after silencing NG2 with siRNA (Fig 3B and 3D). It is noteworthy, that α-secretase activity on NG2_del leads to high levels of the short NG2 CTF (of 12 kD, Fig 4A and 4B, blue) in Oli-neu such that it becomes the major NG2 fragment compared to the NG2 full-length (300 kD) protein (Fig 6A and 6B; [21]). Similarly transfection of the NG2_ICD increases the levels of the NG2 ICD (8.5 kD) such that it becomes the dominant NG2 protein fragment, mimicking γ-secretase activity on the NG2 CTF which releases the endogenous ICD [21]. Taken together both expression constructs markedly increase levels of the small fragments (CTF, ICD) of NG2 compared to the FL protein. Interestingly, PTGDS protein levels were reduced in both cases (NG2_del and NG2_ICD), although the effects were strongest for the NG2_del construct. ADAM 10 Pro form (A10 Pro) was again used as a control. Thus reduction of total NG2 protein but also high expression levels of the short NG2 cleavage fragments, CTF and ICD, reduce PTGDS protein levels.

## Discussion

### NG2-expressing OPC share many characteristics with neurons

Despite the fact that NG2-expressing OPC differentiate primarily into oligodendrocytes, they exhibit several properties typical of neurons. Thus, they express outwardly rectifying sodium channels but in most cases these do not seem to generate action potentials [51]. Additionally, they exhibit bona-fide synaptic structures with neurons in all areas of the brain and at all ages studied (reviewed in [14]): here the NG2 cells constitute a postsynaptic compartment and express a postsynaptic density of slightly less density than classic neuronal PSDs. Our results add the production of neuromodulatory factors to this list of properties that these cells exhibit.

### Expression of Nptx2 by OPC

Nptx2 has been shown to be secreted by neurons in an activity-dependent manner [47]. It binds to the GluR2 subunit of neuronal AMPAR at the cell surface and influences their stability and trafficking [49], subsequently altering neuronal signal conduction [52, 53]. Here we show that primary OPC (pOPC) express Nptx2 at a comparable level to young cultured neurons and that expression is lost with OPC differentiation. In addition, in OPC isolated from total brain by FACS and directly analyzed without an additional culture step, Nptx2 mRNA is enriched in OPC compared to the negative sort fraction of the cells from total brain. Since most neurons do not survive brain dissociation at P9, this negative fraction largely contains immature neurons, microglia, astrocytes and oligodendrocytes. Expression of the NG2 protein had no influence on the expression levels of Nptx2 in OPC, since Nptx2 mRNA was enriched in the same way in FAC-sorted OPC (positive sort) compared to the negative sort fractions from WT and NG2-KO mice. Furthermore, no differences in mRNA levels were observed between FACS-isolated OPC from WT compared to NG2-KO mice. These results demonstrate that OPC contribute to the expression and function of Nptx2 in the CNS, which was to date assumed to be exclusively a neuronal property. Furthermore, they imply that OPC may be able to modulate the neuronal network by releasing Nptx2, with comparable effects to release of Nptx2 from neurons.

### Expression of PTGDS by OPC

Prostaglandin D2 (PGD2) has been associated with multiple CNS functions [48]. The enzyme PTGDS catalyzes the conversion of PGH2 to D2 and thus regulates PGD2 levels in the CNS. PTGDS expression has been demonstrated in oligodendrocytes and the meninges [46]. It is present in the cerebrospinal fluid (CSF), implying secretion [54]. We observed that PTGDS is also expressed by OPC with high expression levels in purified populations of pOPC, interestingly PTGDS and NG2 protein levels were highest at DIV1 within the studied timespan. PTGDS protein levels were also strongly dependent on the state of differentiation of the cells, as they varied greatly depending on the time in culture. Initial reports describing PTGDS expression did not distinguish between OPC and mature oligodendrocytes [46]. We observed that PTGDS mRNA is highly enriched in WT OPC compared to other cells, as seen by cells directly analyzed (but not cultured) after FACS isolation from P9 mouse brain. Furthermore, levels of PTGDS mRNA are highly reduced in OPC from the NG2-KO mouse, but increased in the negative sort fraction. This suggests that there is a compensatory effect in the NG2-KO mice from other cell-types, most likely oligodendrocytes or astrocytes which is supported by unchanged protein levels of both proteins in soluble protein fractions from brain samples of WT and NG2-KO genotypes from P9 mice. The NG2-dependent regulation was confirmed by the complementary approach of silencing NG2 protein expression by siRNA, which also reduced PTGDS protein levels in OPC.

A reduction of PTGDS protein levels was also observed when the intracellular domain of NG2 was expressed in the OPC cell-line Oli-neu, either as an initially membrane bound form (NG2_del) which is then cleaved to yield high levels of CTF and lower levels of the ICD, or as the NG2_ICD which is initially expressed in the cytoplasm but subsequently yields very high levels of nuclear NG2 ICD protein. The strongest reduction of PTGDS levels is observed with expression of NG2_del. These results appear to contradict the reduction in PTGDS expression observed after silencing/knock-out of total NG2 protein levels, but may be explained by the high levels of the small NG2 cleavage fragments (CTF, ICD) compared to the FL protein levels. High CTF levels in relation to reduced FL levels are indeed of physiological relevance directly after cleavage of the FL protein by a α-secretase, which increases the CTF levels as the primary cleavage product [21].

Our results thus show that PTGDS expression is influenced by OPC differentiation, where it correlates with high NG2 protein levels, as well as by different ratios of NG2 cleavage fragments. Taken together, this implies a complex underlying mechanism and suggests an influence of NG2 protein on the expression of PTGDS protein in OPC during differentiation. Furthermore it implies an important role of increased NG2 cleavage as stimulated by neuronal activity [21] or present in lesions [29, 55, 56], as a regulator of PTGDS protein expression levels in OPC.

An increased PTGDS expression has been reported in multiple sclerosis lesions and in human and animal models of demyelination [57, 58] where it seems to be restricted to oligodendrocyte-lineage cells and reactive astrocytes in the lesioned white matter [59]. Thus, under pathological/lesion conditions, reactive astrocytes can also contribute to PTGDS expression [60]. In these studies OPC were not discriminated from oligodendrocyte lineage cells. Our results suggest that OPC should be considered as a source of PTGDS in addition to oligodendrocytes and reactive astrocytes, and also show that the NG2 protein affects levels of PTGDS.

Interestingly a recent study in the PNS demonstrated that the ICD of Neuregulin-1 regulates PTGDS expression levels in dorsal root ganglion neurons (DRGs) [42], correlating with myelination deficits in PTGDS-KO mice. Together with our results presented here, this suggests that PTGDS expression is influenced by several cell-type specific ICDs.

### Role of the NG2 ICD

The Notch protein ICD (NICD) is one of the best characterized ICDs [61] that acts as a transcription factor in the nucleus and contains a conserved DNA binding motif [31]. The NG2 ICD contains no such conserved DNA-binding domain structure, but ELM database sequence analysis revealed in addition to the C-terminal PDZ2 binding motif with known interaction partners [32–34], a WW4 binding motif, as well as two nuclear localization sequences (NLS) at the N-terminus of the ICD. It has been reported that WW domain containing proteins (as potential NG2 ICD interaction partners) locate and signal to the nucleus, in a similar fashion to transcription factors [62]. The NG2 ICD was found in nuclear stainings and biochemical isolates of nuclear fractions, upon transient (over-)expression, suggesting a signaling function in the nucleus. Future studies will reveal nuclear signaling functions of the NG2 ICD and target genes.

## Supporting Information

S1 Fig MACS sorted pOPC. **A:** DIC pictures of sorted pOPC and negative (neg.) sort fraction at three selected time-points of culture (DIV0, 1 and 2; scale bar = 50μm). **B** pOPC at DIV1 stained with antibodies used for identification of the neural cell-types shown in Fig 1B. Neurovascular cells (pericytes) defined as NG2+/SMA+ cells were present in the mixed cultures in C but were not detected in pOPC. Only a very few round SMA+ cells were detected in the pOPC and had the appearance of unspecifically stained dead cells (scale bar = 40μm). **C** Test stainings of primary cultures of cortex from E15 brains with the staining conditions used for the sorted pOPC cultures, here NG2+/SMA+ pericytes could be identified as large amorphic cells (scale bar = 40μm). E = embryonic day. SMA: smooth muscle actin.
(TIF)

S2 Fig NG2 construct localization. **A:** Standard epi-fluorescent pictures of NG2_del_Myc (red) show strong staining at the plasma-membrane (compare endo NG2, green) and intracellular membranes, but nuclei are spared. Expression of the NG2 ICD (NG2_ICD_Myc) results in an almost homogeneous cytoplasmic staining including an intense staining of the nuclei (scale bar = 10μm). **B:** Transfected pOPC showed a much lower expression of the NG2 constructs. Transfection of a GFP plasmid was used for assessing transfection efficiency. After subtracting the signals from the primary Myc AB (Myc pAB) control for the Myc channel, only a strong staining close to the nucleus in NG2_del_Myc transfected cells remained. In NG2_ICD_myc-transfected cells, many cells additionally showed a staining of nuclear substructures shown in more detail in Fig 3C. Images show the maximum projection of an entire confocal z-stack (scale bar = 30μm). (A, 100% of the NG2_del and over 70% of the NG2_ICD transfected cells exhibited the described effects).
(TIF)
