# Supplementary figures and images for: Oligodendrocyte Precursor Cells Synthesize Neuromodulatory Factors
Source: PLoS One. 2015 May 12;10(5):e0127222. doi: 10.1371/journal.pone.0127222 (PMC4429067; doi:10.1371/journal.pone.0127222)

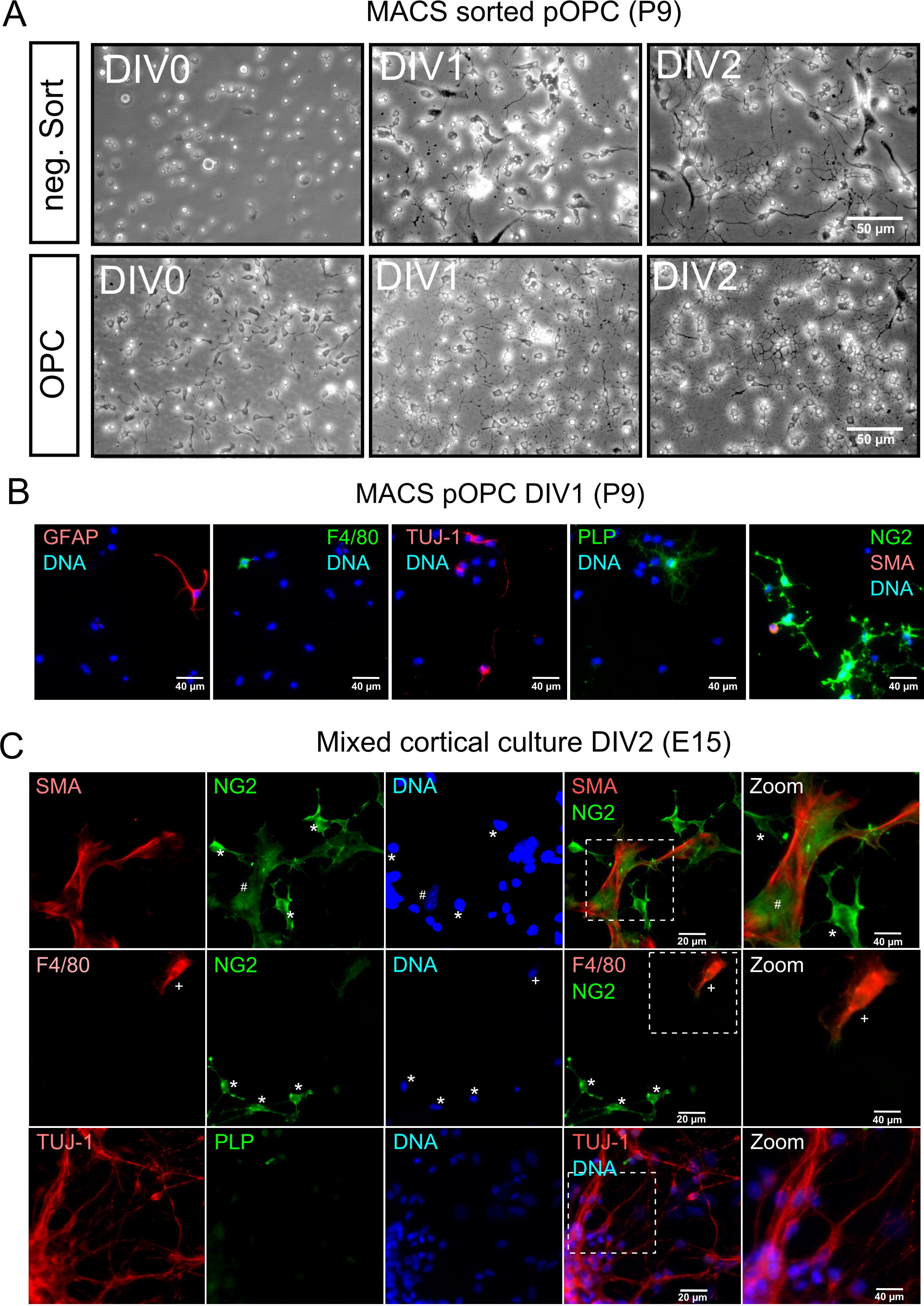

Supplement: S1 Fig — A: DIC pictures of sorted pOPC and negative (neg.) sort fraction at three selected time-points of culture (DIV0, 1 and 2; scale bar = 50μm). B pOPC at DIV1 stained with antibodies used for identification of the neural cell-types shown in Fig 1B. Neurovascular cells (pericytes) defined as NG2+/SMA+ cells were present in the mixed cultures in C but were not detected in pOPC. Only a very few round SMA+ cells were detected in the pOPC and had the appearance of unspecifically stained dead cells (scale bar = 40μm). C Test stainings of primary cultures of cortex from E15 brains with the staining conditions used for the sorted pOPC cultures, here NG2+/SMA+ pericytes could be identified as large amorphic cells (scale bar = 40μm). E = embryonic day. SMA: smooth muscle actin. (TIF) [file pone.0127222.s001.tif]

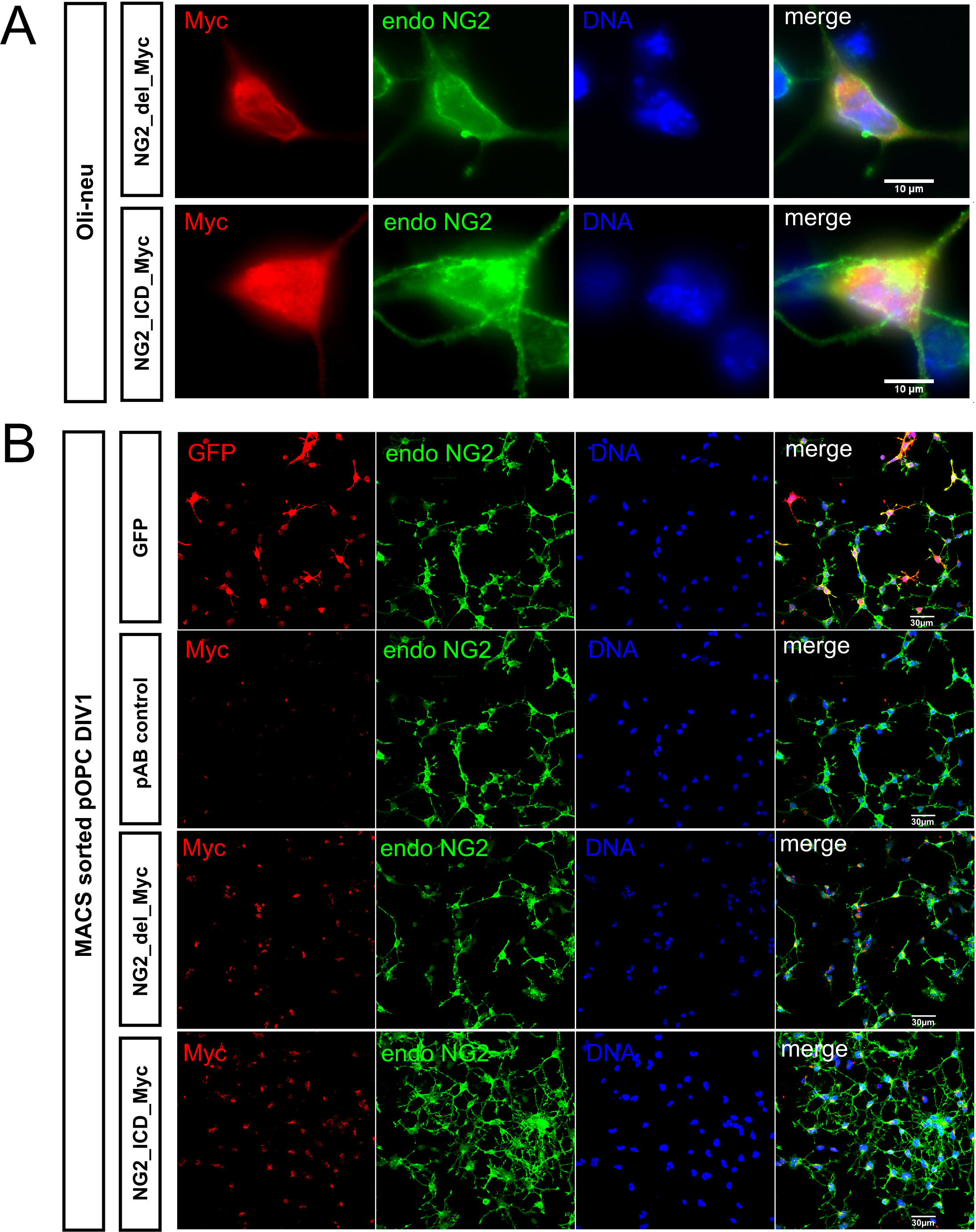

Supplement: S2 Fig — A: Standard epi-fluorescent pictures of NG2_del_Myc (red) show strong staining at the plasma-membrane (compare endo NG2, green) and intracellular membranes, but nuclei are spared. Expression of the NG2 ICD (NG2_ICD_Myc) results in an almost homogeneous cytoplasmic staining including an intense staining of the nuclei (scale bar = 10μm). B: Transfected pOPC showed a much lower expression of the NG2 constructs. Transfection of a GFP plasmid was used for assessing transfection efficiency. After subtracting the signals from the primary Myc AB (Myc pAB) control for the Myc channel, only a strong staining close to the nucleus in NG2_del_Myc transfected cells remained. In NG2_ICD_myc-transfected cells, many cells additionally showed a staining of nuclear substructures shown in more detail in Fig 3C. Images show the maximum projection of an entire confocal z-stack (scale bar = 30μm). (A, 100% of the NG2_del and over 70% of the NG2_ICD transfected cells exhibited the described effects). (TIF) [file pone.0127222.s002.tif]
